# Assessing Choline, Carnitine, and Betaine Intake and Their Effects on Trimethylamine N-Oxide Levels: Validation of a Dietary Questionnaire in a Central European Population

**DOI:** 10.3390/nu17142263

**Published:** 2025-07-09

**Authors:** Witold Streb, Anna Olma, Mateusz Pajor, Alex Suchodolski, Wiktoria Staśkiewicz-Bartecka, Anita Stanjek-Cichoracka, Katarzyna Mitręga, Jacek Kowalczyk, Zbigniew Kalarus

**Affiliations:** 1 Department of Cardiology and Electrotherapy, Faculty of Medical Sciences in Zabrze, Medical University of Silesia, ul. M. Curie-Skłodowskiej 9, 41-800 Zabrze, Poland; 2 Silesian Centre for Heart Diseases in Zabrze, 41-800 Zabrze, Poland; 3 Department of Food Technology and Quality Assessment, School of Public Health in Bytom, Medical University of Silesia in Katowice, 41-808 Zabrze, Poland; 4 Department of Biophysics, Faculty of Pharmaceutical Sciences in Sosnowiec, Medical University of Silesia, 41-200 Sosnowiec, Poland

**Keywords:** trimethylamine N-oxide (TMAO), choline, carnitine, betaine, nutrition questionnaire, validation of dietary tools

## Abstract

Background/Objectives: Trimethylamine N-oxide (TMAO) is implicated in the development of atherosclerosis and cardiovascular diseases. Preventive strategies must recognize the excessive consumption of products rich in choline, carnitine, and betaine, which are substrates essential for TMAO synthesis. The aim of this study was to develop and validate a dietary questionnaire to assess the consumption of these compounds and investigate the correlation with serum TMAO levels in a Central European population. Methods: A dietary questionnaire was designed based on a literature review identifying foods high in TMAO precursors. The tool was validated in a prospective study with 94 participants. The theoretical relevance and reliability of the tool were assessed using factor analysis and statistical indices. Reproducibility was evaluated in a subgroup of 10 participants who completed the questionnaire a second time 24 h later. The results of the questionnaire helped us to determine factors contributing to serum TMAO levels. Results: The final questionnaire consisted of 15 questions, providing acceptable data quality (KMO = 0.654). Three main dietary factors were detected: (1) the consumption of fish products and legumes (SS loadings = 1.72; 10.78% variance), (2) the consumption of cereal products and root vegetables (SS loadings = 1.61; 10.05% variance), and (3) the consumption of meat (SS loadings = 1.47; 9.22% variance). Conclusions: The validated questionnaire is a useful tool for assessing the intake of TMAO-promoting foods in post-myocardial infarction patients from Central Europe. It may support dietary risk assessment and nutritional counseling in clinical practice, particularly for secondary cardiovascular prevention.

## 1. Introduction

Trimethylamine N-oxide (TMAO) plays a crucial role in the development of atherosclerosis, acting as a pro-inflammatory metabolite associated with diet, gut microbiota, and the metabolism of phospholipids and choline. Increased blood levels of TMAO have been shown to correlate with a higher risk of cardiovascular events, such as myocardial infarction and stroke [1,2]. Tang et al. showed that participants who experienced major adverse cardiovascular events during 3-year follow-up exhibited significantly higher baseline plasma TMAO concentrations compared to those without events (median: 5.0 μM [IQR: 3.0–8.8] vs. 3.5 μM [IQR: 2.4–5.9]; *p* < 0.001). Moreover, individuals in the highest quartile of TMAO levels had more than a twofold increased risk of experiencing a cardiovascular event compared to those in the lowest quartile (hazard ratio: 2.54; 95% CI: 1.96–3.28; *p* < 0.001) [3].

**Figure 1:**
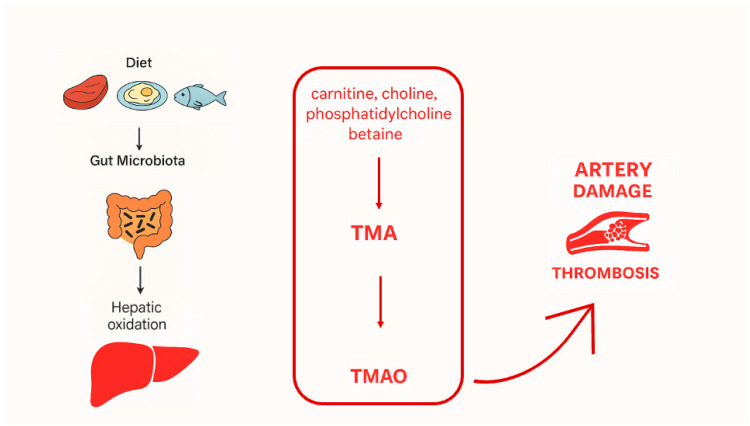
Mechanism linking diet, TMAO, and vascular damage.

TMAO is a metabolite produced from the dietary consumption of choline-, betaine-, or carnitine-rich foods, such as red meat, fish, and eggs. Initially, these precursors are converted to trimethylamine (TMA) by gut microbiota, followed by the hepatic oxidation of TMA to TMAO, a process catalyzed by enzymes in the flavin monooxygenase family (Figure 1) [4].

TMAO has been implicated in the pathogenesis of atherosclerosis through various mechanisms [5]. One suggested pathway involves the downregulation of hepatic cholesterol transport receptors, such as the LDL receptor, which reduces hepatic cholesterol uptake and promotes lipid deposition in vascular walls. Additionally, TMAO inhibits the reverse cholesterol transport pathway from macrophages to high-density lipoprotein, thereby encouraging the formation of foam cells and the development of atherosclerotic plaques [6].

TMAO directly impairs vascular endothelial cells, increases vascular permeability, and leads to monocyte adhesion to the endothelium, thereby triggering inflammatory responses. Additionally, TMAO exacerbates inflammation by stimulating the activity of macrophages and promoting their differentiation into a pro-inflammatory M1 phenotype, accompanied by the elevated secretion of cytokines such as interleukin-6, interleukin-1β, and tumor necrosis factor-alpha. Studies by Seldin et al. (2016) [7] have elucidated the pivotal role of gut microbiota-derived TMAO in promoting vascular inflammation and thrombosis [7]. In mouse models, dietary supplementation with TMAO led specifically to the upregulation of pro-inflammatory signaling pathways, particularly MAPK and NF-κB, resulting in endothelial activation and vascular inflammation. Mice fed a choline-enriched diet exhibited a significantly increased expression of key inflammatory mediators, including monocyte chemoattractant protein-1 (MCP-1), macrophage inflammatory protein-2 (MIP-2), tumor necrosis factor-α (TNF-α), intercellular adhesion molecule-1 (ICAM-1), keratinocyte-derived chemokine (KC), cyclooxygenase-2 (COX-2), E-selectin, and vascular cell adhesion molecule-1 (VCAM-1). Additionally, CD68, a macrophage marker, was upregulated, further supporting the pro-inflammatory and pro-atherogenic effects of TMAO-generating dietary components.

Furthermore, TMAO intensifies platelet hyperactivity, raising the risk of thrombosis [8]. Recent research has identified a direct role of gut microbiota-derived TMAO in modulating platelet function and enhancing thrombotic risk. In a cohort of over 4000 individuals, elevated plasma TMAO levels were shown to independently predict the 3-year risk of thrombotic events, including myocardial infarction and stroke. Laboratory studies revealed that direct exposure of platelets to TMAO enhances stimulus-dependent activation—particularly under submaximal stimulation—by increasing intracellular calcium release, a key step in platelet aggregation pathways [9].

Dietary interventions and the modulation of gut microbiota are proposed strategies for reducing TMAO-related cardiovascular risk. However, limiting the nutritional precursors of TMAO—specifically choline, carnitine, and betaine—remains controversial, as these compounds play essential physiological roles. Current guidelines recommend a daily intake of 425 mg of choline for adult women and 550 mg for adult men [10]. Carnitine requirements are estimated at 15 mg per day, primarily sourced from endogenous production and additional dietary sources. A typical omnivorous diet provides about 24–145 mg of carnitine daily, while vegan diets supply only around 1.2 mg [11]. In contrast, betaine offers beneficial effects when consumed in amounts ranging from 0.5 to 2.5 g each day [12]. Nevertheless, excessive dietary intake of these precursors has been linked to increased serum TMAO levels, which heighten the risk of atherosclerosis progression [13,14].

Despite the clinical importance of TMAO and its dietary precursors, no validated tools currently exist in clinical practice to assess how frequently foods rich in choline, carnitine, and betaine are consumed.

The authors of the present study aimed to develop and validate a dietary questionnaire to assess the frequency of consumption of foods rich in choline, carnitine, and betaine in relation to serum TMAO concentrations within a Central European population.

## 2. Materials and Methods

Information on foods rich in choline, carnitine, and betaine was compiled from the existing literature (Table 1). Based on this information, an initial version of a paper-based dietary questionnaire was developed. The questionnaire utilized a six-point Likert scale to assess the frequency of consumption of specific foods. The scale was defined as follows: 0—never or less than once per month, 1—one to three times per month, 2—once per week, 3—two to four times per week, 4—once per day, 5—two to three times per day, and 6—four or more times per day.

Respondents were instructed to indicate their average consumption of each food item over the preceding year. The validation study for the nutrition questionnaire was designed as a prospective, single-center investigation. Ethical approval was obtained from the Bioethics Committee of the Silesian Medical University in Katowice. Participants were recruited consecutively among patients admitted to the hospital with a diagnosis of acute myocardial infarction. After reviewing the study protocol and providing informed consent, patients completed the dietary questionnaire and provided blood samples for determining TMAO levels. All study procedures were conducted between the second and fourth day of hospitalization. The sample size was determined based on a 10% margin of error and a 95% confidence level. Since the required sample size does not change significantly for populations larger than 20,000, this value was used as the assumed population size. In the absence of prior information about the distribution of responses to individual questions, a 50% response distribution was assumed, which yields the largest required sample size. A total of 94 patients participated in this study, with 10 patients completing the questionnaire a second time 24 h after the initial administration to assess test–retest reliability.

The validation process began with an evaluation of the internal correlations of questionnaire items using Kendall’s tau correlation coefficient. The sphericity of the correlation matrix was subsequently assessed using Bartlett’s sphericity test. Sampling adequacy for the questionnaire was evaluated using the Kaiser–Meyer–Olkin (KMO) test. The theoretical validity of the questionnaire was examined through exploratory factor analysis, employing the principal components method with simple oblimin rotation and Kaiser normalization. Based on the Kaiser criterion, principal components with eigenvalues greater than 1 were retained for interpretation. The reliability of the questionnaire was assessed using Cronbach’s alpha to measure internal consistency.

The reproducibility of the questionnaire for assessing the consumption of TMAO-elevating foods was examined using Cohen’s Kappa between responses from the two measurements. This step ensured the stability and reliability of the tool across repeated administrations.

The determination of TMAO was carried out using Nexera X2 ultra-high-performance liquid chromatography in combination with an LCMS-8045 triple quadrupole (Shimadzu). Samples were prepared by mixing 80 µL of methanol containing the internal standard (TMAO-d9, 25 ng/mL), 10 µL of the sample, and 10 µL of ultrapure water in an Eppendorf-type tube (0.6 mL). The mixture was incubated for 10 min in a thermomixer (ThermoMixer C, Eppendorf) at 20 °C at 2000 rpm, followed by centrifugation for 15 min at 12,000 rpm at 4 °C. The resulting supernatant was transferred to chromatography dishes with a glass insert, and 3 µL of the sample was injected into the chromatography column. Blank and control samples were prepared for quality control. External (trimethylamine N-oxide, 95%) and internal (trimethylamine-d9 N-oxide, 98%) standards were used for the assays, ensuring high sensitivity and specificity of the analysis.

## 3. Results

The developed dietary questionnaire, based on information regarding choline, carnitine, and betaine content, initially consisted of 28 items. Intercorrelation analysis among items addressing the consumption of specific food groups revealed only weak correlations (Table S1). Bartlett’s test of sphericity yielded statistically significant results (χ^2^ = 1347, *p* < 0.01), indicating sufficiently strong correlations among variables and thus justifying the application of factor analysis.

The KMO measure of sampling adequacy for the initial set of items was 0.497, reflecting low sampling adequacy. Consequently, poorly correlated items were removed. The final questionnaire comprised 15 items, with an improved KMO score of 0.654, indicating acceptable data quality for conducting factor analysis (Table 2).

Initial eigenvalues suggested the presence of three factors meeting the Kaiser criterion (eigenvalues > 1), supporting the consideration of a three-factor structure. Variable assignment to factors was based on dominant factor loadings, where loadings > 0.4, a standard threshold in factor analysis, were deemed indicative of significant associations between variables and factors (Table 3). Uniqueness values reflected the proportion of variance in each variable that was not explained by the factors. The scree plot confirmed an inflection point after three factors, further supporting the three-factor solution (Figure 1). The sum of squared loadings (SS loadings) exceeded 1 for each factor, with Factor 1 contributing 1.72 (10.78% variance), Factor 2 contributing 1.61 (10.05% variance), and Factor 3 contributing 1.47 (9.22% variance).

Low inter-factor correlations confirmed the factors’ independence, enhancing the interpretation’s validity. The correlation coefficients between Factor 1 and Factors 2 and 3 were 0.155 and 0.249, respectively, while the correlation coefficient between Factors 2 and 3 was 0.11.

The extracted factors were interpreted as follows:

- Factor 1: Dominant consumption of fish products and legumes.

- Factor 2: Consumption of cereal-based products and root vegetables (e.g., beets and quinoa).

- Factor 3: Consumption primarily associated with meat products (e.g., beef, veal, and ready-to-eat meat dishes).

The analysis suggests that the identified factors effectively described food consumption patterns within the studied sample. Variables with low KMO values (e.g., veal and ready-to-eat meat dishes) may be less helpful in assessing TMAO precursor intake. The factor loadings highlight the clear grouping of food items into three major categories.

In the next validation step, reliability analysis was performed using Cronbach’s alpha to evaluate the internal consistency of the questionnaire. The obtained Cronbach’s alpha value was 0.71, falling within the acceptable range (≥0.7). This result indicates sufficient consistency of the developed tool for assessing the consumption of TMAO precursor-rich foods. Detailed results, including the impact of individual items on the overall score, are presented in Table 4.

Repeatability testing showed that the questionnaire was generally repeatable. Most questions achieved high agreement (≥80%), indicating robust agreement between answers given at different times. Cohen’s Kappa for most questions was in the range of 0.5–1.0, indicating moderate to excellent agreement (Table 5).

TMAO concentrations according to the declared frequency of consumption of each food group are shown in Table 6. The analysis shows that animal products such as fish (both freshwater and marine), poultry, beef, and processed meat dishes had the most significant effect on TMAO concentrations in the study population. Increases in TMAO concentrations were also observed with some plant products, such as beetroot and groats, but their impact was less pronounced than that of meat products. The results confirm that consuming products rich in TMAO precursors, such as carnitine and choline, mainly contained in meat, contributes more to the increase in TMAO levels than plant products.

Interestingly, the intake of some products that have shown a strong association with TMAO concentrations in other studies, such as seafood, was low in our study population, which may have limited their impact on the observed results. Similarly, plant products such as soya, which shows a potential protective effect by lowering TMAO levels, were consumed less frequently. This suggests that the diet of our study population was largely based on meat products, which may have contributed to the overall increase in TMAO concentrations.

**Figure 2:**
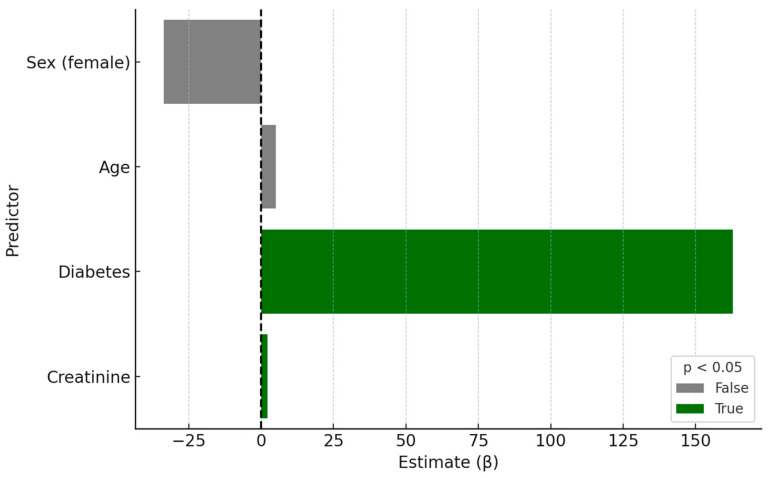
Regression coefficients for TMAO.

In a multivariable regression model excluding GFR, both diabetes and serum creatinine were found to be significant predictors of plasma TMAO concentration (Figure 2). Individuals with diabetes had significantly higher TMAO levels (β = 162.86, *p* = 0.027), and serum creatinine was positively associated with TMAO (β = 2.24, *p* = 0.046). Age and sex did not show statistically significant effects. The model explained approximately 19.1% of the variance in TMAO concentrations (*R*^2^ = 0.191).

## 4. Discussion

Dietary assessment questionnaires continue to pose challenges in clinical practice and research. Several factors may influence the distortions arising from their use and the subsequent misinterpretation of results. One aspect addressed is the impact of cognitive function on response errors [15]. In turn, Dwyer et al. (1997) demonstrated that while reliability data are often regarded as accuracy estimates, such data provide information only on relative intake. They may be helpful for an initial assessment of association with disease but do not serve as a basis for determining absolute intake levels [16].

Archer et al. (2018) highlighted additional issues related to using questionnaires for assessing diet, including logical errors (such as categorization and reification), reliance on human memory and recall, measurement errors stemming from self-reported data, and the pseudo-quantification of qualitative data [17]. Studies involving individuals with obesity have identified inter-individual variability in the under-reporting of calorie and other nutrient intake, which weakens the link between diet and disease [18]. Furthermore, different individuals may interpret questions in various ways, resulting in diverse responses.

Challenges in designing dietary questionnaires also stem from the complexities of nutritional interactions, including the seasonal availability of food products. Additionally, constructing a valid dietary questionnaire must consider various factors related not only to the categories of food products or the characteristics of the population for which they are intended but also to its validation [19]. This necessity arises, among other reasons, from the regional specificity of dietary patterns and demographic variables such as age and education level. Cultural influences and religion may also shape dietary choices. Tailoring dietary questionnaires to the unique characteristics of target groups while considering dietary differences based on residence and cultural or religious beliefs is crucial for obtaining accurate and reliable data regarding the consumption of products that impact TMAO levels.

Fish is established as the primary product rich in TMAO. Consuming fish leads to an immediate increase in plasma TMAO concentration, distinguishing it from other foods. However, the TMAO content in fish varies depending on the species and their habitat. High TMAO levels have been observed in deep-sea fish, such as halibut, cod, herring, and haddock. In contrast, freshwater fish such as zander and trout, along with some marine fish such as tuna, generally have lower concentrations of this compound.

Furthermore, the spike in plasma TMAO levels after eating fish is short-lived, raising questions about its potential long-term effects on the severity of atherosclerotic lesions [20,21]. The impact of fish on serum TMAO levels also depends on regional consumption patterns. The types of fish available in a region, their regular consumption, and portion sizes are critical factors.

However, Andraos et al. (2020) demonstrated in an Australian population that, in addition to fish, the consumption of red and processed meats significantly influenced TMAO concentrations in adults. In contrast, poultry consumption had a clear impact on children [22]. Similarly, the Carlsruhe Metabolomics and Nutrition (KarMeN) study conducted in Germany identified fish and red meat as potential sources of TMAO, although the explained variance in TMAO levels associated with their consumption was minimal [23]. The associations between TMAO concentrations and the consumption of eggs and dairy were less definitive. The effects of these foods may depend on factors such as gut microbiota composition, cooking methods, and food processing, all of which are influenced by cultural and regional context [24]. These factors are also influenced by region.

This suggests that consuming foods high in TMAO or its precursors may result in variations in serum TMAO levels influenced by geographical and cultural factors. This specificity necessitates the development of diverse tools to evaluate the impact of diet on TMAO concentrations across different populations, considering local dietary customs and the availability of certain products.

Gut microbiota composition plays a key role in converting TMAO precursors, such as choline and L-carnitine, into trimethylamine (TMA), which is metabolized to TMAO in the liver. In populations with different dietary habits, gut microbiota composition can vary significantly, affecting TMAO levels in the body. Research indicates that dietary differences can influence the composition of the gut microbiota, which in turn determines the efficiency of this conversion. A plant-based, fiber-rich diet can modulate gut microbiota composition, reducing TMAO production even when precursors are ingested. Studies suggest that dietary interventions using soluble fiber can lower TMA and TMAO metabolism by 40.6% and 62.6%, respectively [25].

Furthermore, studies show that the mere presence of genes associated with TMAO production in the microbiota does not always correlate with the actual metabolic activity of these bacteria. This indicates that the gut microbiota composition does not always directly determine its ability to produce TMAO after consuming specific dietary components [26]. Therefore, regional dietary differences can impact gut microbiota composition, subsequently influencing TMAO production in the body. Understanding these relationships is crucial for developing effective dietary strategies to modulate TMAO levels and associated potential health risks.

Research by Yoo W. et al. (2021) has demonstrated that a diet high in saturated fat can alter gut microbiota composition, favoring the growth of bacteria that produce TMA, thereby increasing TMAO levels in the body. Saturated fats found in products such as red meat, fatty dairy products, and fast food can change the microbiota to promote the growth of bacteria that metabolize choline and L-carnitine into TMA. This may explain the weaker effect of protein-rich plant-based products, such as soy, on TMAO concentrations [27]. Moreover, in our study, the impact of plant products on TMAO concentrations was significantly lower than that of meat products. In this context, it can be interpreted that diets rich in saturated fat and processed meats are well-established risk factors for elevated TMAO concentrations, in part due to their influence on gut microbial composition.

Although we did not conduct direct microbiome profiling, the role of microbial variability in modulating TMAO production cannot be overlooked. Large-scale metagenomic studies, such as the MetaHIT [28] and COMETS [29] consortia, have shown significant interindividual differences in the abundance of TMA-producing bacteria, such as members of the Clostridia and Enterobacteriaceae families. These differences can lead to varying metabolic responses to similar dietary patterns [30], underscoring the complexity of diet–microbiota–host interactions. By situating our findings within this broader framework, we emphasize that the observed associations between dietary intake and TMAO concentrations may be mediated, at least in part, by underlying differences in microbiota composition and function.

The presented dietary questionnaire has been proven to be a valuable tool for assessing the intake of products rich in TMAO precursors such as carnitine, choline, and betaine. It was explicitly designed to differ from comprehensive dietary assessment tools such as the EPIC-FFQ and the KarMeN FFQ [31,32]. While those instruments aim to estimate overall nutrient intake across broad populations, our FFQ was developed with a targeted focus: to quantify the intake of foods that are potential precursors of TMAO. In particular, the questionnaire includes a detailed breakdown of food groups relevant to TMAO metabolism, such as freshwater and saltwater fish, egg-based dishes, organ meats, and processed meats rich in choline and carnitine. These food categories are not disaggregated with comparable granularity in existing validated FFQs.

Moreover, while the KarMeN FFQ excludes individuals with chronic diseases and the EPIC cohort was not designed with cardiovascular patients in mind, our tool was developed and pre-tested in a population of patients after myocardial infarction. This is of particular importance, as post-MI individuals may exhibit both distinct dietary behaviors and unique motivations for dietary change, making their assessment needs different from those of the general population. Such adaptation enhances the relevance of the tool for cardiovascular nutrition research and secondary prevention.

In terms of structure, the EPIC-FFQ is a retrospective 12-month questionnaire comprising over 200 items, which poses a considerable burden for respondents. In contrast, our FFQ is both retrospective and annual in scope but has been streamlined in length and complexity, improving its feasibility in clinical and outpatient settings. Compared to KarMeN, which uses a shorter 30-day recall period and a simplified item list, our tool strikes a balance between temporal depth and practical usability—retaining sufficient dietary time coverage while remaining concise enough for use in routine dietary screening or research settings.

To our knowledge, none of the existing validated FFQs have been explicitly designed or calibrated to estimate intake of TMAO precursors. This represents a key methodological innovation, especially considering the growing interest in TMAO as a potential biomarker and mediator in cardiometabolic disease.

Furthermore, the tool may serve as a practical tool for the dietary estimation of choline, betaine, and carnitine intake, particularly when combined with reference metabolomic data. This creates opportunities for integrated analyses linking habitual diet, metabolite profiles, and clinical outcomes—especially in Central European populations, where dietary patterns differ markedly from those in Western cohorts.

The analysis results indicate that animal products, especially fish, poultry, beef, and processed meat dishes, play a key role in increasing TMAO levels in the body. At the same time, plant products containing TMAO precursors show a lesser effect on TMAO concentrations.

Significant differences in TMAO levels were observed based on the frequency of consumption of each food group, highlighting the utility of the questionnaire in monitoring diet and its relationship with TMAO metabolism. The variations in diet within the study population suggest that personalized dietary interventions can be employed to manage TMAO levels, which may have significant implications for the prevention and treatment of cardiometabolic diseases.

In conclusion, the dietary questionnaire, in combination with TMAO determinations, can provide a practical and sensitive tool for assessing dietary effects on metabolism and metabolic health. The findings highlight the need for further studies on the impact of diet on TMAO levels, considering both animal and plant-based products, to develop optimal dietary recommendations in clinical practice.

Given the key physiological role of choline, carnitine, and betaine, long-term prospective studies are warranted to clarify the potential cardiovascular risks associated with chronically elevated intake of foods rich in these compounds. Although the presented questionnaire has been validated in a Central European cohort, its broader applicability requires replication in populations with diverse geographic, cultural, and dietary backgrounds. Such validation would allow for assessment of its versatility and potential limitations across varying nutritional environments and food availability.

To further improve dietary assessment accuracy and overcome limitations inherent to memory-based, self-reported instruments, future development may benefit from integrating novel digital technologies. In the context of evolving nutritional science and digital health tools, the incorporation of artificial intelligence (AI) and the Internet of Things (IoT) offers considerable potential for advancing both assessment and personalized dietary interventions, particularly in post-myocardial infarction patients. The presented questionnaire could, in the future, be complemented by IoT-based food tracking systems—such as connected kitchen scales, barcode scanning apps, or smart refrigerators—capable of objectively quantifying the intake of TMAO precursors in grams. This would enable real-time monitoring of dietary behavior and may help mitigate the recall bias and inaccuracies associated with traditional FFQs [33].

In parallel, AI algorithms could be employed to analyze large-scale dietary datasets collected via digital FFQs, mobile applications, or wearable sensors, identifying individual nutritional patterns associated with elevated TMAO levels and generating automated, personalized feedback. Such platforms may be calibrated to address multiple cardiometabolic risk factors, including LDL cholesterol, body weight, and insulin resistance, alongside TMAO modulation [34,35]. Moreover, AI-powered virtual nutrition assistants, incorporating machine learning and natural language processing, have already demonstrated potential to enhance adherence to cardioprotective dietary patterns by delivering real-time educational and motivational support [35].

When combined with metabolomic and microbiome data, AI-enhanced dietary tools could contribute to the development of precision nutrition strategies tailored to individual gut microbial capacity for TMAO synthesis. This may be particularly relevant in populations characterized by high interindividual variation in microbiota composition and dietary exposure. Such integration holds promise not only for supporting clinical decision-making but also for informing public health strategies aimed at reducing TMAO and mitigating cardiometabolic risk [36].

A further limitation of the present study lies in the characteristics of the investigated population, which comprised patients hospitalized due to acute myocardial infarction. Individuals in this group may exhibit elevated TMAO levels resulting not only from recent dietary patterns but also from metabolic shifts associated with acute coronary events. This could affect both the progression and destabilization of atherosclerotic plaques. As such, the findings presented here may not be fully generalizable to the healthy population or individuals without established cardiovascular risk and should therefore be interpreted with caution.

Nonetheless, the food frequency questionnaire developed in this study may serve as a practical tool for identifying dietary patterns associated with elevated TMAO concentrations in clinical settings. Its primary application lies in individualized nutritional counseling and cardiovascular risk stratification among patients with existing disease, where modification of TMAO-producing dietary components may support secondary prevention efforts. While further validation in larger and more diverse populations is necessary, the tool may also prove useful for population-level monitoring of dietary behaviors related to TMAO metabolism and associated health risks.

Lastly, the relatively small sample size employed in the test–retest reliability assessment limits the generalizability of the reproducibility findings. Future studies should aim to replicate this analysis in larger and more demographically diverse cohorts to confirm the temporal stability and broader applicability of the questionnaire.

## 5. Conclusions

The presented dietary questionnaire, in combination with TMAO determinations, can provide a practical and sensitive tool for assessing dietary effects on metabolism and metabolic health. The findings highlight the need for further studies on the impact of diet on TMAO levels, considering both animal and plant-based products, to develop optimal dietary recommendations in clinical practice.

## Figures and Tables

**Table 1:** Products rich in TMAO precursors.

Products Rich in Choline/Portion	mg	Betaine-Rich Products/Portion	mg	Products Rich in Carnitine/Portion	mg
Pan-fried beef liver, 100 g	356	Wheat bran, 100 g	1506	Beef steak, cooked, 100 g	42–122
Hard-boiled egg, 1 large	147	Wheat sprouts, 100 g	1395	Milk, whole, 1 glass	8
Beef, lean, braised, 100 g	117	Goi fruit, 100 g	870–1390	Cod, cooked, 100 g	3–5
Soya, ½ cup	107	Spinach, 100 g	725	Chicken breast, cooked, 100 g	2–4
Chicken breast, roasted, 100 g	72	Preserved beetroot, 100 g	333	Cheddar, 33 g	2
Fish, cod, Atlantic, cooked, dry, 100 g	71	Prawns, 100 g	246	Whole-grain bread, 2 slices	0.2
Potatoes, red, roasted, flesh and skin, 1 large potato	57	Wheat bread, 100 g	227	Asparagus, cooked, ½ cup	0.1
Wheat germ, toasted, 100 g	51	Mussels, 100 g	225		
Beans, cashew, can, ½ cup	45	Raw beetroot, 100 g	129		
Quinoa, cooked, 1 cup	43				
Milk, 1% fat, 1 cup	43				
Fat-free vanilla yoghurt, 1 cup	38				
Brussel sprouts, cooked, ½ cup	32				
Broccoli, chopped, cooked, drained, ½ cup	31				
Cottage cheese, fat-free, 1 glass	26				
Fish, albacore tuna, canned in water, drained, 100 g	25				

Source: compiled from 7–9.

**Table 2:** KMO measure of sampling adequacy.

	MSA
Overall	0.654
Freshwater fish	0.554
Saltwater fish	0.710
Seafood	0.596
Canned fish	0.746
Poultry	0.642
Beef	0.601
Veal	0.465
Prepared meat dishes	0.474
Soya products	0.722
Legumes, e.g., broad beans, beans, peas, chickpeas	0.748
Grains, e.g., pumpkin, sesame, sunflower, wheat germ	0.698
Nuts, e.g., peanuts, hazelnuts, walnuts, almonds, pistachios, cashews, coconuts, chestnuts, nut butters/creams	0.665
Spinach	0.700
Red beets	0.662
Coarse groats (buckwheat, buckwheat, brown rice)	0.653
Cooked quinoa	0.610

**Table 3:** Exploratory factor analysis.

Dietary Component	Factor			Uniqueness
	1	2	3	
Legumes, e.g., broad beans, beans, peas, chickpeas	0.571			0.578
Freshwater fish	0.538			0.669
Canned fish	0.531			0.645
Seafood	0.450			0.691
Spinach	0.444			0.729
Beef				0.907
Prepared meat dishes				0.947
Red beet		0.594		0.654
Poultry		0.527		0.694
Soya products		0.478		0.675
Cooked quinoa		0.465		0.718
Coarse groats (buckwheat, buckwheat, brown rice)				0.871
Veal				0.858
Nuts, e.g., peanuts, hazelnuts, walnuts, almonds, pistachios, cashews, coconuts, chestnuts, nut butters/creams			0.852	0.287
Grains, e.g., pumpkin, sesame, sunflower, wheat germ			0.521	0.581
Saltwater fish			0.352	0.691

The ‘minimum residual’ extraction method was used in combination with an ‘oblimin’ rotation.

**Table 4:** Cronbach’s alpha reliability analysis.

Dietary Component	Mean	SD	Cronbach’s α
Freshwater fish	0.6333	0.915	0.705
Saltwater fish	1.2333	1.036	0.674
Seafood	0.0556	0.274	0.705
Canned fish	1.1667	1.081	0.692
Poultry	0.6556	0.886	0.696
Beef	0.7889	0.915	0.701
Veal	0.2556	0.550	0.709
Prepared meat dishes	0.5000	0.808	0.706
Soya products	0.1222	0.469	0.697
Legumes, e.g., broad beans, beans, peas, chickpeas	0.9222	0.960	0.676
Grains, e.g., pumpkin, sesame, sunflower, wheat germ	0.9889	1.237	0.675
Nuts, e.g., peanuts, hazelnuts, walnuts, almonds, pistachios, cashews, coconuts, chestnuts, nut butters/creams	1.3556	1.413	0.691
Spinach	0.6778	0.816	0.691
Red beets	1.6000	1.012	0.704
Coarse groats (buckwheat, buckwheat, brown rice)	1.1556	1.035	0.703
Cooked quinoa	0.0222	0.148	0.709

**Table 5:** Evaluation of the reproducibility of the nutrition questionnaire using the Cohen Kappa method.

	Agreement %	Kappa	Z	*p* Value
Q1	90	0.818	3.02	0.003
Q2	90	0.855	4.42	<0.001
Q3	80	0.636	2.46	0.014
Q4	100	1.000	4.3	<0.001
Q5	100	1.000	3.16	0.002
Q6	80	0.701	3.71	<0.001
Q7	70	0.531	2.82	0.005
Q8	60	0.375	2.05	0.041
Q9	80	0.286	2.11	0.035
Q10	70	0.565	3.27	0.001
Q11	50	0.367	0.238	0.017
Q12	80	0.747	5.02	<0.001
Q13	80	0.692	3.43	<0.001
Q14	90	0.615	2.11	0.035
Q15	70	0.524	2.35	0.019
Q16	90	0.615	2.11	0.035

**Table 6:** Median TMAO concentration according to dietary component.

Dietary Component	0	1	2	3	4	5	6	χ^2^	df	*p*
	Median TMAO Concentration									
Freshwater fish	286	284	200	474	-	-	-	9.95	3	0.019
Saltwater fish	322	270	263	348	323	-	-	9.76	4	0.045
Seafood	286	415	-	-	-	-	-	0.0349	1	0.852
Canned fish	310	276	349	202	-	-	-	7.4	3	0.060
Poultry	145	164	289	321	647	-	-	33.1	4	<0.001
Beef	297	284	226	337	-	-	-	8.56	3	0.036
Veal	289	264	741	-	-	-	-	5.1	2	0.078
Prepared meat dishes	322	267	431	256	132	-	-	14.8	4	0.005
Soya	295	214	-	-	-	-	-	7.65	1	0.006
Legume seeds	337	276	284	317	-	-	-	7.14	3	0.068
Grains, e.g., pumpkin, sesame, sunflower, wheat germ	295	276	229	311	335	-	-	5.91	4	0.206
Nuts	293	263	390	265	260	-	-	12.8	4	0.012
Spinach	297	276	285	354	-	-	-	1.44	3	0.696
Beetroot	245	276	295	355	119	-	-	25.4	4	<0.001
Coarse cereals	309	254	335	380	-	132	-	11.4	4	0.022
Fine groats	255	322	280	297	770	-	-	16.1	4	0.003
Quinoa	286	-	-	-	-	-	-	Na	Na	Na

## Data Availability

The data are available in the Supplementary Data Files.

## Supplementary Materials

The following supporting information can be downloaded at https://www.mdpi.com/article/10.3390/nu17142263/s1, Figure S1: Scree plot; Table S1: Intercorrelations between the different questions of the original version of the questionnaire.

## Abbreviations

The following abbreviations are used in this manuscript:

KMO	Kaiser–Meyer–Olkin test
TMA	Trimethylamine
TMAO	Trimethylamine N-oxide
