# Supplementary material for: Assessing Choline, Carnitine, and Betaine Intake and Their Effects on Trimethylamine N-Oxide Levels: Validation of a Dietary Questionnaire in a Central European Population
Source: Nutrients. 2025 Jul 9;17(14):2263. doi: 10.3390/nu17142263 (PMC12300400; doi:10.3390/nu17142263)
Supplement: Supplementary file 1 [file nutrients-17-02263-s001.zip › nutrients-3689583-supplementary.pdf]

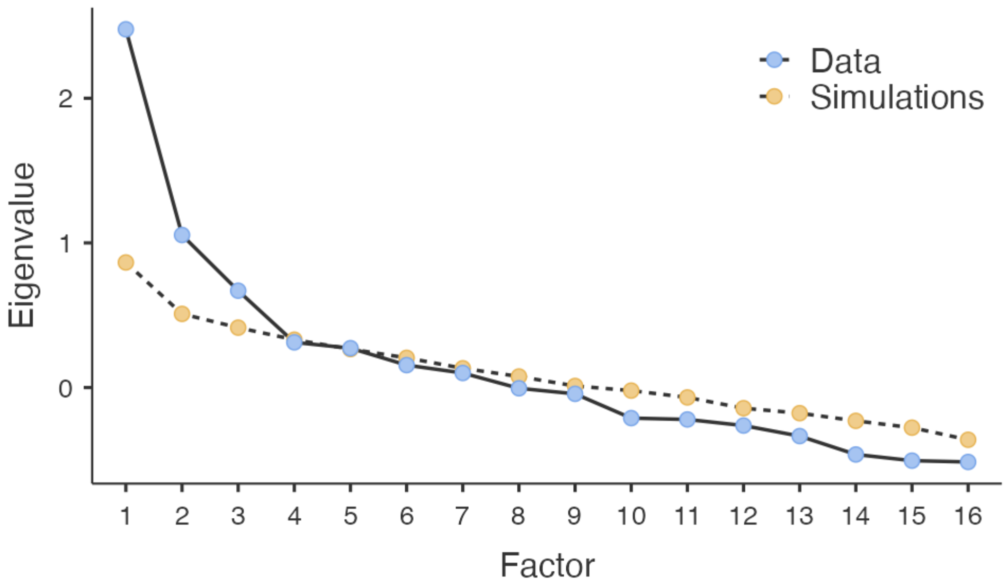

Figure S1. Scree Plot.

Table S1. Intercorelations between the components of the questionnaire

	Q1	Q2	Q3	Q4	Q5	Q6	Q7	Q8	Q9	Q10	Q11	Q12	Q13	Q14
Q1	–													
Q2	0.158	–												
Q3	0.215	0.012	–											
Q4	0.214	0.110	0.183	–										
Q5	0.120	0.249	0.023	0.137	–									
Q6	0.009	0.016	-0.029	-0.013	0.100	–								
Q7	-0.085	0.222	0.075	0.156	0.115	0.005	–							
Q8	-0.018	-0.021	-0.092	0.032	0.098	0.055	-0.039	–						
Q9	0.009	0.112	0.118	0.095	-0.232	0.047	-0.007	-0.158	–					
Q10	-0.096	0.078	0.103	-0.009	0.106	0.040	0.041	0.060	0.201	–				
Q11	0.004	0.025	-0.064	0.038	0.078	0.058	0.042	0.303	0.029	0.059	–			
Q12	-0.012	0.015	0.225	0.121	-0.080	-0.121	0.103	0.133	0.248	-0.053	0.106	–		
Q13	-0.025	0.173	-0.068	0.168	0.047	-0.120	0.157	-0.041	0.038	0.179	-0.032	0.112	–	
Q14	0.151	0.311	0.174	0.289	0.058	-0.145	0.091	0.038	0.121	-0.171	0.161	0.203	0.135	–
Q15	-0.016	0.297	0.288	0.120	-0.014	0.092	0.143	-0.006	0.096	0.102	-0.014	0.117	0.169	0.317
Q16	0.052	0.291	0.226	0.036	0.015	-0.045	0.057	0.009	0.210	0.169	0.048	0.207	0.007	0.190
Q17	-0.071	-0.064	-0.018	-0.102	0.098	0.008	-0.058	0.149	-0.128	0.069	0.176	0.121	-0.004	-0.010
Q18	0.160	0.170	0.089	0.247	0.080	0.065	0.205	-0.081	0.167	-0.018	0.030	0.130	0.242	0.232
Q19	-0.088	0.171	-0.211	0.128	0.094	0.192	0.149	-0.015	0.083	0.171	0.159	-0.050	0.209	0.088
Q20	-0.093	-0.118	-0.141	-0.129	0.076	0.164	-0.143	0.084	-0.113	-0.103	0.136	-0.175	-0.127	-0.109
Q21	0.045	0.098	-0.041	-0.038	0.050	-0.072	0.020	-0.003	-0.133	-0.082	0.023	0.049	0.044	0.135
Q22	0.162	0.155	0.127	0.110	0.012	0.026	0.155	-0.148	0.006	0.018	-0.146	0.036	0.170	0.121
Q23	-0.101	0.018	-0.050	0.015	0.056	0.000	0.046	0.081	-0.003	-0.126	0.181	0.052	0.025	0.067
Q24	-0.120	0.171	-0.036	0.037	0.079	-0.174	0.247	-0.194	0.074	0.137	-0.157	-0.106	0.226	0.050
Q25	0.097	0.023	0.215	-0.010	-0.058	0.055	0.054	-0.116	0.125	-0.055	-0.083	0.116	-0.036	0.023
Q26	0.025	-0.168	0.193	-0.124	0.051	0.189	-0.091	0.036	0.005	0.055	-0.055	0.025	-0.125	-0.092
Q27	0.084	-0.080	-0.051	0.070	0.140	-0.073	0.032	0.142	-0.021	0.019	0.189	0.112	-0.126	0.093
Q28	0.201	0.110	0.200	0.034	0.115	-0.027	-0.005	-0.010	-0.025	0.076	0.010	0.110	-0.135	0.034

	Q15	Q16	Q17	Q18	Q19	Q20	Q21	Q22	Q23	Q24	Q25	Q26	Q27	Q28
Q15	–													
Q16	0.442	–												
Q17	-0.109	-0.134	–											
Q18	0.177	0.104	-0.038	–										
Q19	0.041	-0.009	0.044	0.101	–									
Q20	-0.244	-0.213	0.328	-0.151	-0.102	–								
Q21	0.118	0.198	-0.026	0.130	0.030	-0.401	–							
Q22	0.172	0.093	-0.027	0.161	0.145	-0.241	0.134	–						
Q23	0.129	0.016	0.188	0.070	0.205	0.124	0.019	0.133	–					
Q24	0.171	0.148	-0.184	-0.016	0.159	-0.181	-0.003	0.180	0.111	–				
Q25	-0.013	0.021	0.147	0.134	-0.004	0.126	-0.034	-0.031	-0.027	-0.173	–			
Q26	0.008	0.058	0.177	0.063	-0.066	0.130	-0.099	0.047	0.002	-0.155	0.179	–		
Q27	0.016	-0.020	0.165	-0.130	-0.002	0.231	-0.027	-0.069	-0.028	-0.154	0.158	0.038	–	
Q28	0.014	0.127	0.186	0.075	0.005	0.003	0.073	0.078	0.111	-0.119	0.231	0.241	0.206	–
